# Enteritis in raccoons (*Procyon lotor*) caused by an infection with zoonotic *Salmonella* and carnivore parvovirus

**DOI:** 10.1186/s12917-025-04560-y

**Published:** 2025-02-24

**Authors:** Kristin Pütsch, Ingo Spitzbarth, Regina Scheller, Kristin Heenemann, Florian Hansmann

**Affiliations:** 1 Institute of Veterinary Pathology, Faculty of Veterinary Medicine, Leipzig University, An den Tierkliniken 33, Leipzig, 04103 Germany; 2 Saxon State Laboratory of Health and Veterinary Affairs, Bahnhofstrasse 58-65, Leipzig, 04158 Germany; 3 Institute of Virology, Faculty of Veterinary Medicine, Leipzig University, An den Tierkliniken 29, Leipzig, 04103 Germany

**Keywords:** Enteritis, Salmonellosis, Parvovirosis, Raccoon, Germany

## Abstract

**Background:**

The raccoon (*Procyon lotor*) is a potential carrier of a large number of zoonotic pathogens, and its population is increasing in urban areas in Europe. In the present study, we investigated two cases of fatal enteritis in raccoons in Germany. Parvoviruses are a common cause of enteritis in raccoons, however in these cases an additional infection with zoonotic *Salmonella* was found, which has not yet been described in other countries than the United States.

**Case presentation:**

Two female raccoons, aged 14 and 18 weeks, were submitted for necropsy. Histopathology of the small intestine revealed crypt degeneration and necrosis, atrophy and fusion of villi, as well as numerous bacteria partially covered by fibrinous pseudomembranes. By microbiological culture of small intestinal samples *Salmonella enterica *subsp*. enterica *Serovar Kottbus and *Salmonella enterica *subsp.* enterica *Serovar Ferruch were isolated, respectively. In addition, *carnivore* *protoparvovirus type 1* was identified in the small intestine of both animals.

**Conclusions:**

The infection of raccoons with carnivore *protoparvovirus type 1* results in immunosuppression, which facilitates the spread of other pathogens. Both isolated *Salmonella* serovars represent a significant zoonotic threat for humans being in contact with the raccoon. Furthermore, in raccoons with sudden death a double infection with *carnivore protoparvovirus type 1* and *Salmonella* should be considered as an important differential diagnosis.

## Background

Raccoons, representing one of the invasive species in Europe, are highly susceptible to several pathogens, including rabies virus*,* canine distemper virus, and *Baylisascaris procyonis*. Consequently domestic animals, captive animal populations as well as native species are endangered and partly their diseases entail zoonotic potential [1–4]. Another hazard, particularly in relation to juvenile animals is the *carnivore protoparvovirus type 1* (CPPV-1) [5]. Due to the immunosuppressive effect of parvoviruses, secondary infections frequently occur [6, 7]. Parvoviruses are single-stranded DNA viruses that cause a spectrum of disease patterns in different animal species. Parvoviruses preferentially infect mitotically active cells such as intestinal epithelial cells, whereby the transferrin receptor type-1 is decisive for cell entry [8]. Previously reported co-infections in raccoons include *Anaplasma phagocytolyticum* and *Cryptosporidium* spp. as well as canine distemper virus [2, 9]. Nevertheless, at present only one description of a dual infection with *Salmonella enterica* subsp. *enterica* exists in the USA, while no case has been described in Europe so far [10]. *Salmonella* are gram-negative *Enterobacteriaceae* predominantly associated with infections of the gastrointestinal tract, but also septicemia and abortions can occur [11]. Studies in other countries revealed that *Salmonella* serovars, being isolated from raccoons, are likely to be pathogenic to humans and showing a single or multiple antimicrobial resistance [12]. In the present study, two raccoons from an animal sanctuary that exhibited a short period of enteritis followed by sudden death were investigated.

## Case presentation

Two female 14- and 18-weeks old wild-living raccoons taken by an animal sanctuary were submitted for necropsy after a short period of apathy to clarify, whether an infectious cause of disease was present. No further clinical signs were observed prior to death in the animal sanctuary for injured or ill raccoons. Health care of the juvenile raccoons included vaccination against common diseases of dogs, like hepatitis contagiosa canis, leptospirosis and canine parvovirosis. Additional biosecurity measures at the animal sanctuary included quarantine of all new animals for a duration of two weeks as well as regular hand and surface disinfection procedures. Especially during quarantine health condition (including urination, defecation, respiration, food and water consumption as well as behavior) were checked multiple times a day. General virological and microbiological screening methods for newly introduced animals are not carried out.

**Fig. 1:**
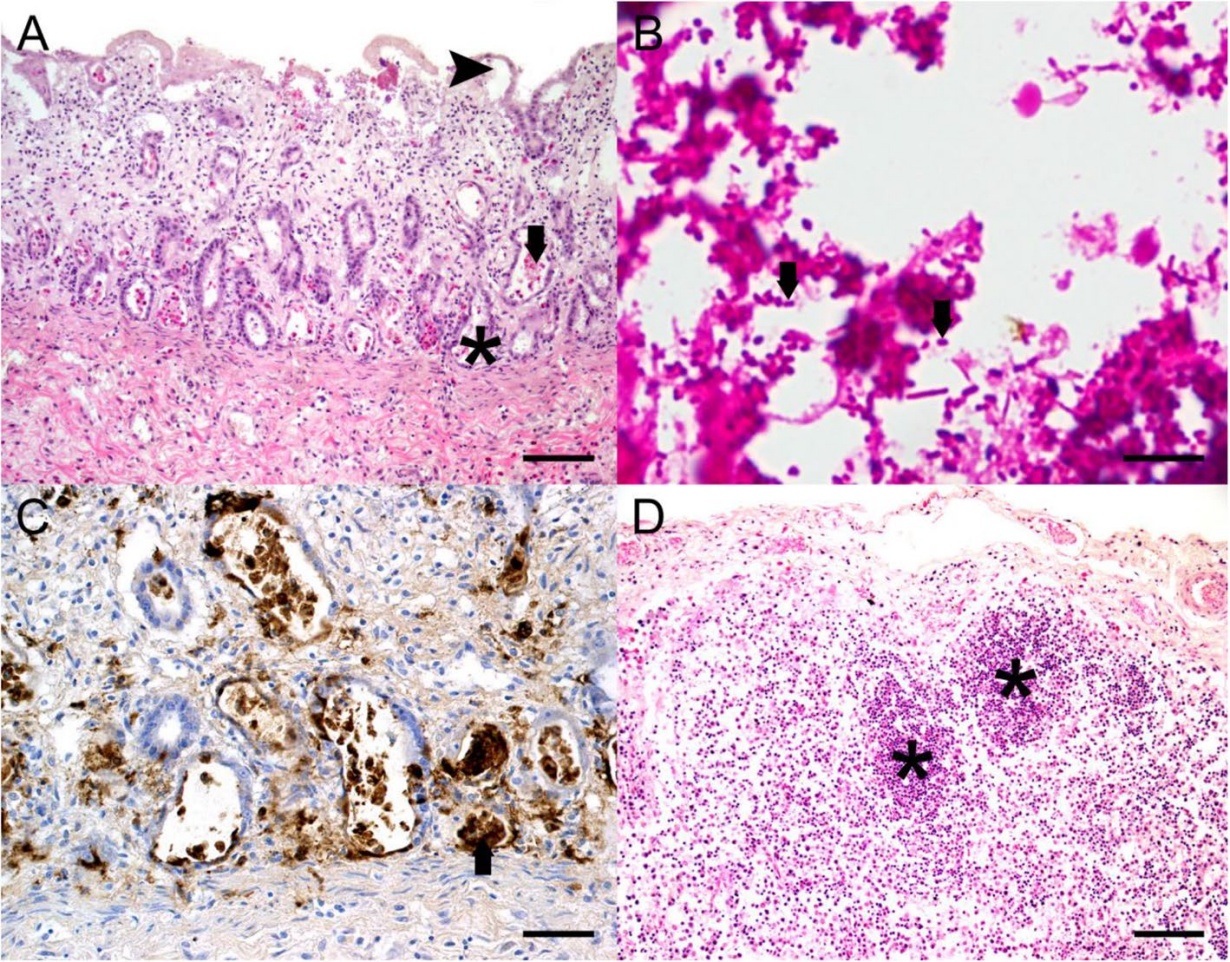
**A** The small intestine showed a severe, diffuse, lymphohistioplasmacytic, partly fibrinous-necrotising enteritis with fusion and atrophy of villi (  ), crypt abscesses (  ), crypt dilatation (  ) and untypical crypt regenerates (HE, bar indicates 100 µm). **B** In the small intestine numerous intralesional and intraluminal gram-negative bacteria (  ) were visible (gram staining, bar indicates 20 µm). **C** Parvovirus immunohistochemistry visualized parvo-viral antigen (  ) in intestinal crypt cells (bar indicates 50 µm). **D** Lymph follicles (  ) were depleted in the mesenteric lymph nodes (HE, bar indicates 100 µm)

Gross lesions in both raccoons were restricted to a moderate to severe, diffuse, catarrhal, partly fibrinous enteritis. Samples from several organs including the intestines, mesenteric lymph nodes, spleen and bone marrow were taken and fixed in 10% neutral buffered formalin overnight followed by dehydration and embedding in paraffin wax. After sectioning, 2 µm thick slices were stained with hematoxylin and eosin (HE). Histopathological examination of the intestines revealed lymphohistioplasmacytic, partly fibrinous-necrotising enteritis with fusion and atrophy of villi, crypt abscesses, crypt dilatation, and atypical crypt regeneration (Fig. 1A). Furthermore, intralesional accumulations of rod-shaped bacteria were visible. Gram staining demonstrated numerous intraluminal and intralesional gram-negative bacteria (Fig. 1B).

Histopathological findings were suggestive of an underlying parvovirus infection. Therefore, immunohistochemistry was performed on tissue sections of the small intestine, bone marrow and mesenteric lymph nodes as previously described [13]. Briefly, the endogenous peroxidase activity of the tissue sections mounted on glass sides was blocked with 0,5% H₂O₂ in methanol for 30 min. Subsequent the sections were buffered in tris-buffered-saline (TBS) (pH 7,6), pretreated with protease (0,05%, 5 min, 37 °C) and blocked with normal goat serum in TBS (5%, 20 min). Parvovirus immunohistochemistry was conducted with peroxidase–antiperoxidase (PAP)- method using a primary monoclonal mouse antibody targeting canine/feline parvovirus antigen (monoclonal mouse anti-parvovirus antibody, dilution 1:400, CPV1-2A1, Custom Monoclonals International, Sacramento, California) and a secondary rat-anti-mouse IgG (AffiniPure Rat Anti-Mouse IgG, 1:100, Jackson ImmunoResearch Laboratories, Inc., USA). The antibody binding was visualised using 3,3’-diaminobenzidine-tetrahydrochloride (DAB, Sigma Aldrich, Steinheim, Germany) followed by counterstaining with Mayer’s haematoxylin.

A high frequency of Parvovirus antigen-immunolabeled intestinal crypt epithelial cells compared to a lower number of immunolabeled epithelial cells in the villi was detected (Fig. 1C). Furthermore, the mesenteric lymph nodes showed a moderate to severe, diffuse lymphatic depletion (Fig. 1D), containing numerous immunolabeled lymphoblasts, follicular dendritic cells and macrophages. Lesser numbers of lymphoblasts and haematopetic stem cells in the bone marrow were immunopositive.

Specimens of the small and large intestine as well as mesenteric lymph nodes were submitted to the Saxon State Laboratory of Health and Veterinary Affairs for aerobic, microaerophilic and anaerobic bacterial culture on diverse bovine blood agar plates as well as selective agar plates that were incubated at 37 °C or 40 °C for up to 72 h, and isolates were identified. Salmonella isolates were serotyped using a rapid slide agglutination test and serovars were named according to the Kauffmann-White classification scheme. The microbiological investigations of the intestine identified *Salmonella* Kottbus in the first case and *Salmonella* Ferruch in the second case.

The parvovirus infection was confirmed using PCR targeting parvovirus-specific nucleotide sequences as previously described [5]. For phenotypic analysis the PCR products of the parvovirus-specific-polymerase-chain-reaction were sequenced [14, 15]. Briefly, 1010 base pairs of the isolated nucleotide sequences were compared to reference sequences from the GenBank of the National Center for Biotechnology Information with the help of Maximum-Likelihood-method and 1000 replicates of bootstrap sampling, using Kimura 2-parameter model and MEGA version 7.0 [14]. However, the typing of these viruses was only successful in the second case [16]. Feline panleukopenia virus was revealed and the phylogenetic analysis was presented in a phylogenetic tree.

**Fig. 2:**
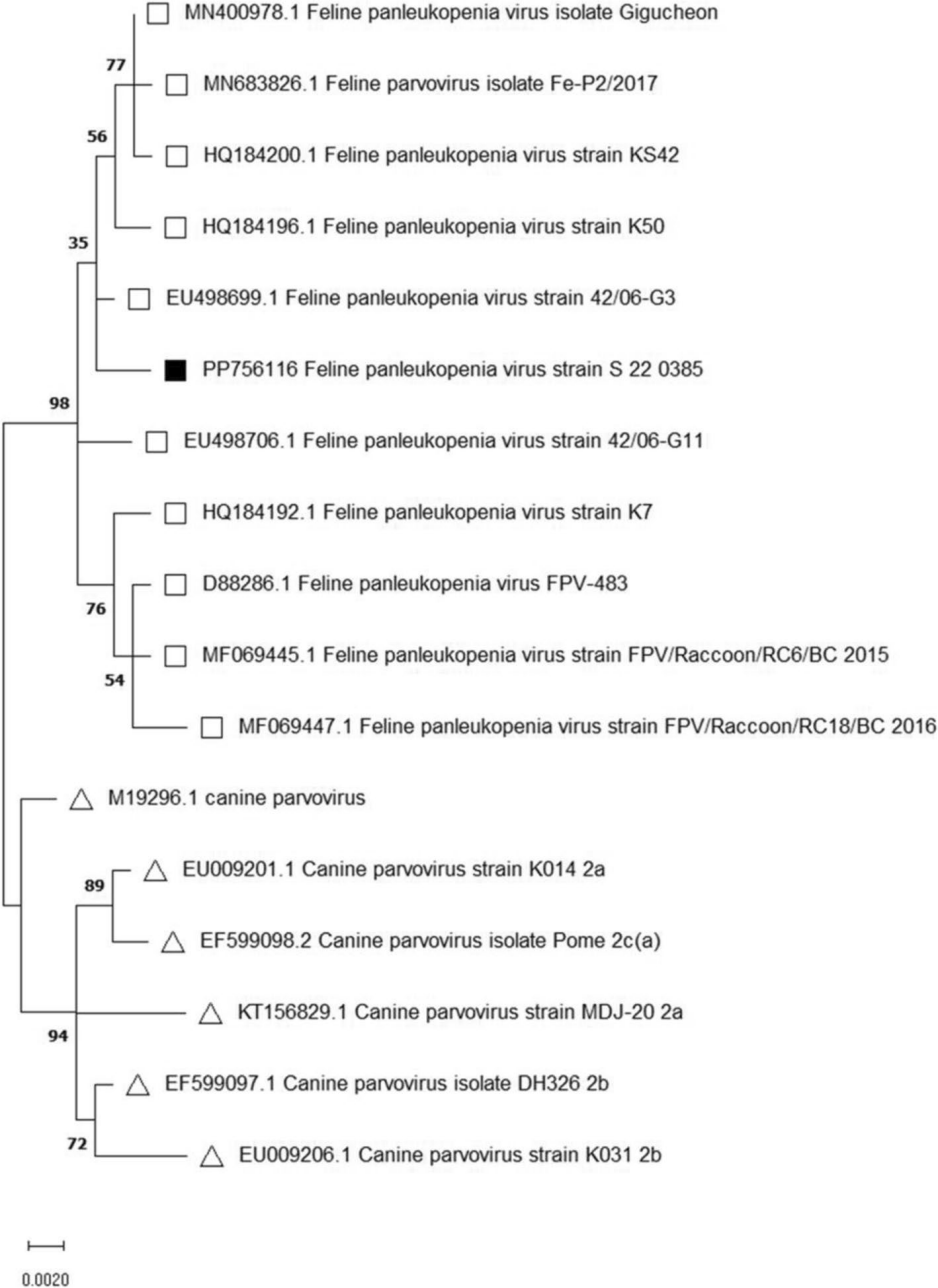
Phylogenetic analysis examining 1010 base pairs of the received sequence from the second raccoon (PP756116 Feline panleukopenia virus strain S 22 0385). The nucleotide sequence was compared to 16 reference sequences listed in the National Center for Biotechnology Information (NCBI). The pedigree was created with the Maximum-Likelihood-method (1000 Bootstrap-repetitions) and the Kimura 2-parameter model using MEGA version 7.0.  sequence from this study (GenBank accession PP756116);  other feline parvoviruses;  canine parvoviruses)

A phylogenetic analysis was conducted using nucleotid sequences to determine the evolutionary relationships between the samples from our raccoons and other known sequences of the *carnivore protoparvovirus Type 1* (Fig. 2). These genomic sequences were compared to related sequences from GenBank using BioEdit and MEGA version 7.0 tools. A phylogenetic tree was constructed with maximum likelihood method with 1000 replicates of bootstrap sampling and the help of the Kimura 2-parameter model using MEGA version 7.0 (Fig. 2).

Accompanying measures in the animal sanctuary included a vaccination of all raccoons using a feline parvovirus vaccine to protect the remaining juvenile raccoons. Following death of the two raccoons cleaning and disinfection were immediately initiated in accordance with the German Veterinary Medicine Society (DVG) guidelines to avoid infections of other animals and humans. No further cases of parvovirus-infections were reported in this animal sanctuary.

## Discussion and conclusions

The potential of raccoons to carry single or multiple zoonotic pathogens as a concern of public health is highlighted [17, 18]. The present study firstly describes a coinfection with *carnivore protoparvovirus type 1* and zoonotic *Salmonella spp*. in raccoons in Europe. CPPV-1 is a worldwide-distributed, opportunistic pathogen of domestic and wildlife animals frequently causing a lethal disease in young animals [19]. Infections in raccoons show similar pathological findings to those observed in cats and dogs, pathologic findings, mainly concerning the intestines, lymphatic system and bone marrow. Nevertheless, an uncommon course of parvovirus infection may occur, for example a parvovirus-induced encephalitis in a raccoon is reported [20]. The frequency of the disease was significantly reduced by the introduction of a regular immunization regime in domestic animals including cats and dogs [21]. Due to the immunosuppressive effect of parvoviruses the occurrence of coinfections is common [6, 7].

In contrast to *canine parvovirus 2* (CPV-2), whose host range is restricted to canines, CPV-2a, 2b and 2c show a more widespread host range also including felines. Consequently, these new subtypes have led to reemerging parvovirus outbreaks even among domestic animals [22]. Not only so far unknown parvovirus-variants represent a threat for susceptible animals, but also reservoirs in wildlife animals can lead to a potentially lethal infection. For example, due to Feline Parvovirus (FPV) wildlife animals like foxes, minks and raccoons are also endangered [5]. Raccoons are highly susceptible to parvoviruses and, as a result, potential transmitters of these viruses, because they can be infected by CPV- and FPV-strains as well as their own raccoon parvovirus (RPV), all belonging to the *carnivore protoparvovirus type 1*. Compared to other species (e. g. red foxes or raccoon dogs) relatively high numbers of parvovirus-infected raccoons in Germany are reported [23]. Beyond that, this invasive species is suspected to be part of the ongoing transmission of parvoviral disease in domestic and nondomestic species [24, 25]. Furthermore, the role in transmission and partly in introducing new genotypes is also described for other pathogens [26, 27].

It is well-known that raccoons are capable of transferring several diseases, which presents a risk to humans, wildlife and domestic animals [1, 3, 12]. Similar cases have been reported from Massachusetts, USA [10]. In these cases, the isolated parvovirus strains were > 99% identical to *Canine Parvovirus-2a* (CPV-2a) reference strains and serotyping revealed a human pathogenic serovar of *Salmonella* with multiple antimicrobial resistances, namely *Salmonella* Thompson*. Salmonella* Ferruch, one of the isolated serovars of *Salmonella enterica* subsp*. enterica* in the present case is also supposed to potentially endanger humans being in contact with affected animals [28]. *Salmonella* Kottbu*s*, the other isolated serovar from our cases, is also known to be a zoonotic serovar of *Salmonella*, which is mainly isolated from poultry samples and potentially carries antimicrobial resistances [29]. The infection of the raccoons with *Salmonella* Kottbus may be attributable to the ingestion of Salmonella infected wild fowl prior to the uptake by the animal sanctuary; for example S. Kottbus was detected in quails and quail eggs in Germany [29, 30]. In addition, at the animal sanctuary both animals were fed raw chicken eggs, which could have potentially served as infection route for *Salmonella* sp. into the animal sanctuary, given the recognized potential of eggs to serve as one of the most important sources of human salmonellosis [31, 32]. Transfer of resistance genes is another hazard in terms of public health accompanied by high prevalences of *Salmonella* among raccoons [10, 33].

The proof of simultaneous infection with *Salmonella* and CPPV-1 leads to the hypothesis that *Salmonella* is widespread among raccoons and primarily causes subclinical infections. Consequently, apart from *Salmonella* infections in livestock animals, which can result in contaminated food products, wildlife animals entail a not to be neglected risk of infection with human-pathogenic *Salmonella*. Several studies have reported a high prevalence of potentially zoonotic bacteria among raccoons [12, 34]. Over the last years the number of raccoons in urban and peri-urban areas in Europe increases [35, 36]. As a consequence, the risk of infection for humans is especially high in urban areas [10, 37]. Investigations in predominantly rural areas of Germany showed that raccoons are more likely to carry *Salmonella* in contrast to other wild carnivores such as foxes and raccoon dogs [23, 38]. Furthermore, phylogenetic analysis of the *Salmonella* isolates revealed close genetic relationship between the *Salmonella* isolates of raccoons and other species (e.g. cattle) [38]. Consequently, raccoons should be considered as important subclinically infected carriers of *Salmonella* bacteria that can shed significant numbers of bacteria if they also suffer from another infection or immunosuppression.

In summary, parvovirus-infections in raccoons should always be taken in consideration in cases of enteritis or sudden death. Due to possible coinfections with e.g. *Salmonella* humans being in contact with raccoons or their samples need to be particularly careful. Since the origin of human diseases is often zoonotic, it is important to understand the ongoing transmission of diseases among wildlife animals, domestic animals, and humans [35].

## Data Availability

The dataset generated and/or analysed during the current study is available in the GenBank repository under the accession number PP756116. All other data obtained or analysed as part of this study are included in this article.

## Abbreviation

HE: Haematoxilin and eosin
